# The diagnostic potential of the iron-regulatory hormone hepcidin

**DOI:** 10.1097/HS9.0000000000000236

**Published:** 2019-06-30

**Authors:** Andrew E. Armitage, Hal Drakesmith

**Affiliations:** 1 MRC Human Immunology Unit, MRC Weatherall Institute of Molecular Medicine, University of Oxford, John Radcliffe Hospital, Oxford, United Kingdom; 2 Haematology Theme, Oxford Biomedical Research Centre, Oxford, United Kingdom

**Take home messages**

- Hepcidin is the central regulator of systemic iron homeostasis, controlling dietary iron uptake and availability of iron for tissues and organs. Hepcidin expression levels are determined by a balance of signals arising from diverse physiological states, ranging from iron and inflammatory status to erythropoietic activity and hypoxia.
- Hepcidin measurement, either as a single index or in concert with other iron-related indices, may aid in diagnosis of iron-related disorders such as iron deficiency (ID), inflammatory anemia, hereditary hemochromatosis and iron-refractory iron deficiency anemia (IRIDA).
- Diagnostic test studies for hepcidin as a single index generally indicate moderate to strong performance in identifying ID, distinguishing iron deficiency anemia from inflammatory anemia, and predicting responsiveness to oral iron interventions.
- Routine implementation of hepcidin assessment in clinical practice and epidemiological surveys will be facilitated by assay standardization, reduced assay cost, application to high-throughput clinical analyzers, and development of robust normal ranges combined with evidence-based guidance in interpretation across clinical/public health settings.

## Introduction

The hormone hepcidin controls dietary uptake and availability of iron. It is regulated by a balance of signals related to iron status, inflammation, erythropoietic activity, and hypoxia, determining iron provision for erythropoiesis and other iron-demanding processes. Progress in understanding hepcidin's involvement in normal physiology and disease conditions,^1^ coupled with advances in quantification, make it an increasingly attractive candidate biomarker for assessing iron status and guiding iron intervention strategies.^2^

## Current state of the art

Hepcidin regulates circulating iron concentrations by triggering ubiquitin-mediated degradation of the iron exporter ferroportin, but also by directly occluding ferroportin-mediated iron transport.^3▪^ Ferroportin is highly expressed by iron-recycling erythrophagocytic macrophages and duodenal enterocytes, so hepcidin activity restricts macrophage iron release and dietary iron uptake, causing hypoferraemia. In contrast, hepcidin suppression increases iron availability for tissues. Recent work also indicates a significant contribution of erythroblast-expressed ferroportin to systemic iron homeostasis.^4▪^

To maintain homeostasis, increases in iron stores and circulating iron are sensed by the liver leading to hepcidin upregulation via the BMP/SMAD signaling pathway. Hepcidin transcription is also directly upregulated during inflammation by JAK/STAT3 signaling. However, it is downregulated during ID, hypoxia and erythropoietic expansion. Expanded erythropoiesis increases expression of the erythropoietin-responsive hormone erythroferrone, which suppresses hepcidin through direct inhibition of BMP6 signaling^5▪^; in addition, erythropoietic activity also consumes iron, further reducing hepcidin stimulatory signals.^6^^,^^7▪^

Failures in hepcidin regulation cause iron-related pathology. Mutations in hepcidin itself, or more frequently in genes encoding components of the upstream BMP-SMAD signaling pathways (eg, *HFE*, *BMP6*, Hemojuvelin *(HJV)*, Transferrin receptor 2 *(TfR2)*) that link iron sensing to hepcidin, result in inappropriately low hepcidin concentrations relative to iron status. These conditions, broadly termed hereditary hemochromatosis (HH), are characterized by toxic iron loading, predominantly affecting liver, pancreas, and heart. Chronically suppressed hepcidin due to ineffective erythropoiesis also explains secondary iron loading in conditions such as beta-thalassemia. In contrast, mutations in *TMPRSS6* (encoding matriptase-2, a negative regulator of hepcidin) cause inappropriately elevated hepcidin, impaired iron absorption and consequent iron-refractory iron deficiency anemia (IRIDA).^1^,^8^

### Hepcidin: a potential iron status biomarker?

ID refers to depletion of total body iron, primarily from reticuloendothelial and hepatic stores. Iron stores depletion leads to iron-restricted erythropoiesis and ultimately to iron deficiency anemia (IDA), its most prominent manifestation.^9^ Importantly, other functional consequences may precede anemia, including impaired brain development in young children.^9^

Absence of stainable reticuloendothelial iron in bone marrow is considered a gold standard means of diagnosing ID; however, being an invasive test, its applicability is limited. Several biochemical markers, together with hematological parameters (eg, MCV, reticulocyte-hemoglobin, hemoglobin), can aid identification of the different stages of iron limitation described above.^9^ Low ferritin can identify depleted iron stores effectively; however, ferritin is also an acute-phase protein switched on during inflammation, meaning it lacks sensitivity when inflammation is present; inflammatory status (eg, concurrent C-reactive protein (CRP) or alpha-1-acid glycoprotein (AGP)) must, therefore, be considered when interpreting ferritin concentrations.^9^ Serum iron or transferrin saturation (Tsat) indicate iron availability for tissues, but demonstrate notable diurnal variation and decrease during inflammation. Iron demand is frequently assessed by quantifying soluble transferrin receptor (sTfR) concentration: during iron-restricted erythropoiesis, iron-deficient erythroblasts increase TfR1 expression, correlating with increased serum concentrations of soluble transferrin receptor (sTfR).

Could hepcidin complement (or even replace) established biomarkers in indicating iron status? Hepcidin can predict response to oral iron,^10^ and so might provide a single-index guide for iron supplementation. Interestingly, the traditionally-used biomarkers could be regarded as proxies for key physiological inputs that control hepcidin expression: stored iron (*proxy = ferritin*) and circulating iron (*proxy = Tsat*) both induce hepcidin, involving BMP/SMAD signaling; inflammation (*proxies = CRP/AGP*) upregulates hepcidin via JAK/STAT3 signaling; iron demand (*proxy = sTfR*) associates with hepcidin suppression via erythroferrone-dependent/-independent BMP pathway restriction. Consistently, serum hepcidin typically correlates positively with ferritin/CRP/AGP, and negatively with sTfR. Moreover, evidence that opposing signals concurrently determine hepcidin expression is accumulating. For example, hepcidin upregulation by acute inflammation can be offset by increased erythroid drive or hypoxia^11^–^14^; enhanced erythropoiesis in beta-thalassemia overrides induction of hepcidin by iron loading; and in inflammatory anemias, chronic inflammation can increase hepcidin, despite the anemia.^1^,^9^ Overall, fine tuning of competing positive and negative signals controls hepcidin synthesis and consequent iron handling. However, for hepcidin to become widely adopted as an indicator of iron status/requirement, this biological appeal must be complemented by robust analytical performance and increased validation of its interpretation across clinical settings.

### Hepcidin as a biomarker: analytical considerations, performance, interpretation

Hepcidin resists freeze-thaw, but adheres to laboratory plastics at room temperature potentially causing concentration underestimation; sample matrix (eg, plasma, serum) may also influence results. Hepcidin, like Tsat, exhibits diurnal variation, which should be accounted for. Several methods for hepcidin quantification are validated: mass spectrometry methods can distinguish hepcidin isoforms, while immunoassays typically offer greater flexibility. However, the various well-performing assays are not currently calibrated equivalently, so although data from different assays correlate well, absolute values returned differ.^15^ When comparing hepcidin data from different studies, knowing which assays were used is essential. Nevertheless, progress towards assay harmonization and ultimately standardization is advancing.^15^,^16^

Several studies have investigated hepcidin's performance as a single index for diagnosing ID, for distinguishing IDA from inflammatory anemia, and for predicting oral iron utilization/response (eg,^17^–^22^ studies reporting ROC^AUC^ with N > 200; see also^2^). While the clinical/epidemiological settings and test definitions used vary considerably, these studies generally indicated moderate to strong test performance, in most cases at least comparable with established tests. Interestingly, independent reports from diverse settings evaluating hepcidin cutoffs for diagnosing ID found very similar optimal cutoffs^19^,^22^ that were also close to a cutoff identifying effective oral iron incorporation into erythrocytes.^19^

## Future perspectives

Hepcidin's inclusion in investigations related to iron is broadening, yet its establishment in routine clinical practice or epidemiological surveys requires several steps. Establishment of standardized reference ranges, implementation on high-throughput analyzers, and reducing assay cost will greatly facilitate its use. A greater breadth of prospective diagnostic test studies ideally with larger sample sizes, testing against gold standard definitions across diverse populations and disease conditions would be desirable. Determining whether a uniform cutoff for effective response to iron interventions exists, and whether hepcidin can indicate efficacy of oral versus intravenous iron interventions across different clinical settings will prove useful.

**Figure 1:**
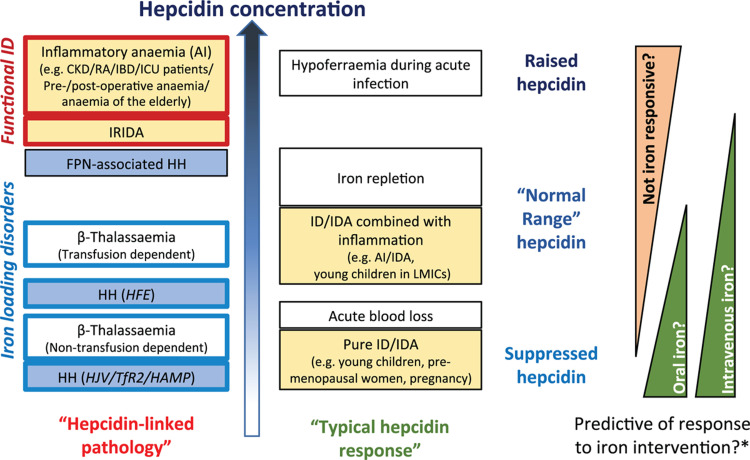
Potential utility of assessing hepcidin concentrations across clinical and physiological settings related to systemic iron homeostasis. Typical hepcidin concentrations vary considerably between diverse iron-related disorders and responses to distinct physiological challenges. Variation in hepcidin may aid differential diagnosis of distinct conditions with similar clinical presentations (here highlighted by fill color: yellow – iron deficiency / anemia / AI; blue – iron-loading disorders). Hepcidin may indicate the likely efficacy of response to iron interventions, and potentially whether oral or intravenous interventions should be preferred (^∗^when iron-overload is not indicated). In the future, hepcidin concentrations may guide the use of hepcidin antagonists (indicated by red outlines to boxes) or agonists (blue outlines) for disordered iron homeostasis. Note: in reality, there is likely considerable overlap in hepcidin concentrations between the conditions shown; the hierarchy shown is not intended to represent an exact order, but to give an indication of typical hepcidin concentrations (ie, typically raised vs normal vs suppressed) across the different conditions. AI = Anemia of Inflammation, CKD = Chronic Kidney Disease, HH = Hereditary Haemochromatosis – (HFE), caused by mutation in the *HFE* gene; (HJV/TfR2/HAMP), caused by mutations in Hemojuvelin, transferrin receptor 2 and hepcidin respectively, IBD = Inflammatory Bowel Disease, ICU = Intensive Care Unit, ID = Iron deficiency, IDA = Iron deficiency anemia, IRIDA = Iron-refractory iron deficiency anemia, LMIC = Low-/Middle-Income Country, NTDT = Non-transfusion dependent beta-Thalassemia, RA = Rheumatoid Arthritis, TDT = transfusion dependent beta-Thalassemia.

Despite diagnostic potential as a single index, clinical interpretation of hepcidin should consider the broader context, including its relationship with concurrent iron and hematological indices. This is especially so for investigations of iron-related disorders caused by inappropriate hepcidin regulation (eg, HH and IRIDA), as discussed above.^1^ Specifically, in the absence of inflammation, low hepcidin/ferritin ratios may contribute to diagnostic work-ups of genetically complex iron-loading disorders including HH,^2^,^23^ while low Tsat/hepcidin ratios may be indicative of IRIDA.^8^ Furthermore, hepcidin may bring diagnostic or disease management benefits in additional contexts besides these (see Fig. 1). In the longer term, hepcidin quantification may guide therapeutic hepcidin agonist and antagonist use in conditions ranging from hereditary hemochromatosis and beta-thalassemia, to inflammatory anemias. Prospective validation of the benefit of adding hepcidin analysis in each of these settings should be prioritized.
